# Effects of Various Drugs on Alcohol-induced Oxidative Stress in the Liver

**DOI:** 10.3390/molecules13092249

**Published:** 2008-09-23

**Authors:** Mira Popovic, Snezana Janicijevic-Hudomal, Biljana Kaurinovic, Julijana Rasic, Svetlana Trivic

**Affiliations:** 1 Department of Chemistry, Faculty of Sciences, Trg Dositeja Obradovica 3, 21000 Novi Sad, Serbia, biljanak@ih.ns.ac.yu (B. K.); 2 Institute of Pharmacology, Faculty of Medicine Pristina (Kosovska Mitrovica), Serbia

**Keywords:** Alcohol (ethanol) stress, liver, bromocriptine, haloperidol, azithromycin

## Abstract

The major aim of this work was to investigate how alcohol-induced oxidative stress in combined chemotherapy changes the metabolic function of the liver in experimental animals. This research was conducted to establish how bromocriptine, haloperidol and azithromycin, applied to the experimental model, affected the antioxidative status of the liver. The following parameters were determined: reduced glutathione, activities of glutathione peroxidase, glutathione reductase, peroxidase, catalase, xanthine oxidase and lipid peroxidation intensity. Alanine transaminase was measured in serum. Alcohol stress (AO group) reduced glutathione and the activity of xanthine oxidase and glutathione peroxidase, but increased catalase and alanine transaminase activity. The best protective effect was achieved with the bromocriptine (AB1 group), while other groups had similar effects on the studied parameters.

## Introduction

A major source of free radicals in biological systems is molecular oxygen (O_2_). By interacting with fundamental cell structures and biomolecules, reactive oxygen species (ROS) can lead to the development of many pathophysiological disorders. Consequently, they are implicated in several pathological disorders, such as ischemia-reperfusion injury, coronary atherosclerosis, diabetes mellitus, hypertension, and cancer genesis, as well as in the aging process [1,2]. Alcohol, the most commonly consumed xenobiotic, generates ROS species whether it is used over a long period of time or whether it is taken acutely in a single large dose. Three metabolic pathways are involved in alcohol metabolism which utilise the following enzymes: alcohol dehydrogenase, the microsomal ethanol oxidative system (MEOS) and catalase. Each of these pathways, in particular MEOS, could produce ROS species. At large concentrations or after long term use, the inducible MEOS pathway (cytochrome P450 2E1) takes over ethanol detoxification [3,4]. Superoxide anion free radicals and H_2_O_2_, which can lead to lipid peroxidation, are produced in this process. MEOS aggravates the oxidative stress directly as well as indirectly by impairing the defense systems. Hydroxyethyl radicals are probably involved in the alkylation of hepatic proteins [5]. Significant amounts of ROS can be generated in the further metabolism of acetaldehyde by aldehyde xantin oxidases [6,7]. Alcohol promotes the generation of ROS and/or interferes with the body’s normal defense mechanisms against these compounds through numerous processes, particularly in the liver. Alcohol reduces the levels of agents that can eliminate ROS (i.e., antioxidants). The resulting state of the cell, known as oxidative stress, can lead to cell injury. ROS production and oxidative stress in liver cells play a central role in the development of alcoholic liver disease [8].

The detrimental effects of alcohol consumption, either acute or chronic, can be potentiated or inhibited with other xenobiotics or drugs. These days large numbers of medications are taken on a daily basis. Many different groups of medications are used, but in practice we do not really know what effects they exert on the parameters of oxidative stress in the liver. Bromcritine, haloperidol and azythromzcin, medications which are not commonly used, have different chemical structures and different pharmacological actions. Despite this they can exert similar or different in the presence of alcohol on biochemical parameters determined in this work.

Bromocriptine (BRC), with potent D_2_ agonistic and mild D_1_ receptor antagonistic action, is widely used in the treatment of Parkinson’s disease. Its mode of action is both central and peripheral and thus is dose dependent with a wide therapeutic window. BRC has recently been shown to possess strong free radical scavenging action *in vitro* and *in vivo* [9]. Yoshikawa [10] in his paper concluded that BRC possesses antioxidant properties. This drug may possibly provide symptomatic benefits in the treatment of Parkinson’s desease.

Haloperidol (HP) is a widely used neuroleptic drug for the treatment of acute and chronic psychosis, e.g., schizophrenia. HP belongs to the butyrophenone group and is thought to exert its clinical effect through cerebral dopamine D2-receptors and δ-receptors. Chronic treatment with neuroleptics increases free radical production and oxidative stress. Chronic use of neuroleptics is also reported to decrease the activity of antioxidant defense enzymes, superoxide dismutase (SOD) and catalase [11]. Yao *et al*. [12] suggested that haloperidol directly or indirectly affects both CAT, superoxide dismutase activity and GSHPx in patients with schizophrenia.

Azithromycin is an antibiotic with immunomodulatory effects. This antibiotic has been successfully used in chronic diseases for periods of six months or more. The side effects of long-term use are known and are mainly limited to gastro-intestinal cramps. The potential for resistance limits its use to individual patients under close supervision. Chia and Chia [13] studied the medical records of chronic fatigue syndrome (CFS) patients for clinical and laboratory data related to the outcome of the treatment with azithromycin. The results of their studies were positive. Bakar *et al*. [14], also showed that azithromycin displays antioxidant effects.

The aim of this work was to investigate the way in which alcohol-induced oxidative stress in combined chemotherapy (bromocriptine, haloperidol and azithromycin) changes the metabolic function of the liver in experimental animals. We therefore measured antioxidative parameters such as catalase activity (CAT, E.C. 1.11.1.6), peroxidase activity (Px, E.C. 1.11.1.7), glutathione peroxidase activity (GSHPx, E.C. 1.11.1.9), glutathione reductase (GR. E.C. 1.6.4.2) and the concentration of reduced glutathione (GSH). In addition we determined the activity of pro-oxidative enzyme xantin oxidase (XOD, E.C. 1.2.3.2) and the level of lipid peroxidation (LPx). The activity of alanine transaminase (ALT, E.C. 2.6.1.2) was also measured as a marker of liver damage, and was correlated with antioxidant enzymes activity changes.

## Results and Discussion

In Table 1 we present values of measured parameters in livers from animals treated with alcohol in combination with chemotherapy with bromocriptnom, haloperidolom and azithromycin.

**Table 1:** Investigated biochemical parameters.

Enzymes	OO group	AO group	AB1 group	AB2 group	AB3 group	AH group	AA group
**GSH**	2.69 ± 0.32	1.41 ± 0.27^c^	1.81 ± 0.11^c, d^	1.12 ± 0.09^c,^ ^d^	1.28 ± 0.14^c^	1.08 ± 0.12 ^c,^ ^d^	0.97 ± 0.10^c,^ ^e^
**GSHPx**	0.96 ± 0.12	0.25 ± 0.05^c^	0.26 ± 0.06^c^	0.55 ± 0.09^c,^ ^f^	0.59 ± 0.11^c,^ ^f^	0.39 ± 0.07 ^c,^ ^e^	0.42 ± 0.06^c,^ ^f^
**GSHR**	2.93 ± 0.22	2.78 ± 0.38	5.74 ± 0.21^c,^ ^f^	3.16 ± 0.25	3.20 ± 0.28	2.78 ± 0.25	2.82 ± 0.19
**Px**	11.36 ± 1.13	8.81 ± 1.26^c^	13.42 ± 1.42^f^	10.74 ± 0.18^e^	12.03 ± 1.22^e^	15.28 ± 1.17^b,^ ^f^	19.01 ± 1.66^c,^ ^f^
**CAT**	4.42 ± 0.32	18.86 ± 1.10^c^	19.74 ± 0.59^c^	13.20 ± 1.23^c,^ ^f^	13.70 ± 0.62^c,^ ^f^	15.17 ± 1.02^c,^ ^f^	16.22 ± 0.67^c,^ ^f^
**XOD**	8.33 ± 0.94	4.30 ± 0.97^c^	3.68 ± 0.19^c^	5.65 ± 0.54 ^c,^ ^d^	2.72 ± 0.29 ^c,^ ^e^	1.90 ± 0.36^c,^ ^f^	4.86 ± 0.60^c^
**LPx**	0.65 ± 0.10	0.51 ± 0.03^a^	0.39 ± 0.09 ^b,^ ^d^	0.35 ± 0.04^c,^ ^f^	0.46 ± 0.02 ^b,^ ^d^	0.47 ± 0.02 ^b,^ ^d^	0.62 ± 0.09^d^
**ALT**	26.8 ± 5.20	45.8 ± 7.50^c^	30.0 ± 4.60^e^	27.60 ± 3.90^f^	41.9 ± 6.50^c^	32.5 ± 4.30^e^	25.9 ± 6.50^f^

Activities of: XOD, CAT, Px , GSHPx and GSHR are expressed in nmol/mg of protein·min^-1^ Intensity of lipid peroxidation is expressed in: nmol malondialdehyde/mg protein Content of GSH is expressed in: nmol GSH/mg protein Activity of ALT are expressed in U/L n=5; compared to OO group: p>0.05 (statistically insignificant), ^a^ p<0.05, ^b^ p<0.01, ^c^ p<0.001 compared to AO group: p>0.05 (statistically insignificant), ^d^ p<0.05 , ^e^ p<0.01, ^f^ p<0.001

The concentration of glutathione was reduced in all groups compared to both controls (OO, AO), except for the AB1 group, which had an increased level of glutathione compared to the AO group. Statistically, the most significant decrease was in AA group compared to both OO and AO groups. Also, a statistically significant decrease in GSH content was observed for the AH group. The activity of glutathione peroxidase was reduced in all groups compared to the OO control, and increased compared to the AO control in all groups except AB1. The activity of glutathione reductase was highest in the AB1 group compared to both of the control groups. The activity of peroxidase was increased only in the AH and AA group compared to the OO control, and increased in all groups compared to the AO control. The activity of xantine oxidase was statistically significantly reduced in all groups compared to the OO control, and significant changes compared to the AO control were noticed in AB2 (increased enzymatic activity), and AB3 and AH (reduced enzymatic activity). The level of lipid peroxidation was reduced in all groups compared to the OO control, except the AA group. Compared to the AO control, AA increased the LPx, and all other groups caused a decrease. The activity of catalase was increased in all groups compared to the OO control, and decreased in all groups compared to the AO control, except in AB1.

Correlation between groups were tested using ANOVA one-way variance analysis as shown in Table 2. Comparison between groups in the determination of GSH content reveals no statistical significance between AB3/AO, AH/AB2 and AA/AH. The activity of GSHPx showed a high level of statistical significance in combination of 19 groups, while only two groups were not statistically significant.

Out of all possible combinations of 21 groups, seven exhibited no statistical significance. Compared to other parameters, alcohol stress and treatment with BCR, HP and AZA had the least effect on the GSHR activity, and the correlation between the groups is the smallest.

Comparison of peroxidase activities between 21 possible combinations of groups using the ANOVA test showed no statistical significance in two combinations (AB2/OO and AB3/OO). Nineteen other combinations of groups exhibited statistically significant correlations.

The activity of catalase is significantly different between all groups except in the case of AB2/AB3. Statistical analysis in all combinations of groups established a significant correlation in XOD activity. The confidence level between combinations of groups was achieved for 16 groups in the determination of levels of lipid peroxidation.

Only the AO and AB3 groups had statistically significant increases in ALT activity compared to the OO group. All groups except AB3 group had a statistically significant decrease of ALT activity comparing to the AO group. ALT is a markers of liver damage. Hence, it may be inferred that acute alcohol stress caused acute liver damage. The AB3 group (a group where BRC had been applied just before the alcohol stress) also showed high ALT activity, while other groups, where drugs had been applied at least 24 hours after the alcoholic stress, had ALT activities similar to OO group, which infers that they displayed protective effect.

In the present investigation we observed that acute alcohol stress led to a decrease in GSH content and the activity of GSHPx, compared with untreated animals (OO group). In combination with the examined drugs, the concentration of GSH was statistically significantly decreased in all groups. The activity of GSHPx was increased in all cases comparing to the AO group, expect in AB1.

**Table 2:** ANOVA test for measured biochemical parameters.

GSH	00	A0	AB1	AB2	AB3	AH	CAT	00	A0	AB1	AB2	AB3	AH
A0	+						A0	+					
AB1	+	+					AB1	+	+				
AB2	+	+	+				AB2	+	+	+			
AB3	+	-	+	+			AB3	+	+	+	-		
AH	+	+	+	-	+		AH	+	+	+	+	+	
AA	+	+	+	+	+	-	AA	+	+	+	+	+	+
**GSHPx**	00	A0	AB1	AB2	AB3	AH	**XOD**	00	A0	AB1	AB2	AB3	AH
A0	+						A0	+					
AB1	+	-					AB1	+	+				
AB2	+	+	+				AB2	+	+	+			
AB3	+	+	+	-			AB3	+	+	+	+		
AH	+	+	+	+	+		AH	+	+	+	+	+	
AA	+	+	+	+	+	-	AA	+	+	+	+	+	+
**GSHR**	00	A0	AB1	AB2	AB3	AH	**LPx**	00	A0	AB1	AB2	AB3	AH
A0	-						A0	+					
AB1	+	+					AB1	+	+				
AB2	+	+	+				AB2	+	+	-			
AB3	+	+	+	-			AB3	+	-	+	+		
AH	-	-	+	+	+		AH	+	-	+	+	-	
AA	-	-	+	+	+	-	AA	-	+	+	+	+	+
**Px**	00	A0	AB1	AB2	AB3	AH							
A0	+												
AB1	+	+											
AB2	-	+	+										
AB3	-	+	+	+									
AH	+	+	+	+	+								
AA	+	+	+	+	+	+							

Results of the ANOVA test are represented for the differences between groups for the confidence level p<0.05.

Compared to the OO group, applied drugs combined with acute alcohol stress lead to a decrease in GSHPx activity. The observed decrease in GSH content, together with the decrease in GSHPx activity could be explained in several ways.

GSH could be conjugated with products of alcohol metabolism, by GSH-S-transferase (but also by nonenzymatic patterns). On the other hand, GSH plays an important role in the protection of the organism against GSH peroxidase activity, which shows that this is one of the ways of defense from the consequences of oxidative stress.

Alcohol stress did not induce notable changes in GSHR activity even in combination with the examined drugs. A single change was induced only in the AB1 group compared with both controls.

Haloperidol administration resulted in a depletion of antioxidant glutathione (GSH) in various regions of the brain in rodents as well as in the CSF of HP-treated patients where this GSH depletion was also associated with an enhanced lipid peroxidation [15]. Yao *et al*. [12], show that haloperidol may not have a direct regulatory effect on the antioxidant defense system enzyme activities and that SOD and GSHPx activities may change in response to other factors.

Reduction of GSH content leads to the accumulation of H_2_O_2_[5], which could explain the high CAT activity observed in this investigation (Table 1). Treatment with bromocriptine, haloperidol and azithromycin leads to decreased CAT activity, compared to stress induced by alcohol (AO group), however, a significant increase in this activity was observed in comparison with the control (OO group). Furthermore, H_2_O_2_ is formed in acute alcohol stress, probably by inclusion of alternative pathways (e.g. MEOS), inducing the activation of CAT and GSHPx.

Lipid peroxidation plays an important role in the induction of cell damage, causing destruction of the lipid membrane. However, in acute alcohol stress, investigated in this work, a decrease in LPx was observed. Furthermore, the combination of drugs with stress induced by alcohol also decreased the activity of LPx. Post *et al*. [15] showed that HP leads to increased LPx, coupled with a lowering of GSH content. One of the triggers for spontaneous lipid peroxidation is a decrease in GSH content (under 1μmol/g liver), a probable cause of the observed insignificant increase in LPx [16]. Many xenobiotics and drugs decrease the intensity of LPx, either by interacting with ROS or by some other pathway [16,17]. We hypothesize that in this investigation a possible explanation may be the interaction of the examined drugs or their downstream metabolites with ROS.

Alcohol stress induces an insignificant reduction in the activity of LPx. However, in combination with the investigated drugs, the activity of LPx was increased, compared to the control groups (AO and OO, respectively). This result is in agreement with both an increase in CAT activity, and reduction in GSH content.

In this investigation, oxidative stress induced by alcohol, and its combination with drugs examined in this study, attenuated the activity of XOD. In addition, the highest decrease was registered in the AB3 and AH group, comparing to both controls. We have also confirmed that XOD generates superoxide radical anion 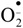 and H_2_O_2_, which are potentially cytotoxic and can lead to lipid peroxidation. Lipid peroxidation is the oxidative conversion of polyunsaturated fatty acids to lipid peroxides, which generate different reactive oxygen species (ROS). ROS and lipid peroxides have been implicated in the pathogenesis of a wide variety of diseases ranging from infectious, inflammatory and autoimmune diseases to atherosclerosis and cancer [18]. Our results point to a reduction of both LPx and XOD activity, probably due to omission of this enzyme in the generation of and other ROS. Furthermore, Aktan *et al*. [18] confirmed a decrease in XOD, after azithromycin treatment.

Acute alcohol stress caused significant changes in the content of GSH, together with changes in the activities of GSHPx, XOD, CAT and LPx.

Our observed results show that the investigated drugs (bromocriptine, haloperidol and azithromycin) in combination with stress induced with alcohol exhibited no “protective effect”. GSH content was decreased in all groups compared to the OO control group. With the exception of the AB1 group, a combination of drugs and alcohol stress decreased GSH content even more than in the AO group. Earlier data [11] showed that chronic treatment with neuroleptics could lead to oxidative stress. However, in this examination HP decreased LPx compared to both controls (OO and AO), together with a reduction of CAT (compared with the AO group). From our results it is obvious that haloperidol exhibits no prooxidant effects. The administration of this drug decreased the glutathione content and increased the activity of LPx in general. Also, the application of HP induced an increase in the GSHPx activity compared to the AO group, and a reduction compared to the OO group. The administration of HP did not exhibit noticeable effects on the activity of GSHR.

Azithromycin is an antibiotic with a wide range of imunomodulatory effects [19,20,21] used in the treatment of number of bacterial infections, due to inhibition of the generation 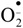 of.

Levert *et al*. [19] show that azithromycin inhibits the generation of O_2_ in the presence of agents which stimulate neutrophile oxidative burst. Consequently, azithromycin exhibits a strong anti-inflammatory and antioxidative effect with very low IC50 values.

From the obtained results it could be concluded that azithromycin is the most powerful drug, which causes changes in all investigated parameters compared to the AO group, and possibly leads to increased damage induced by alcohol.

Azithromycin is the only drug which caused an increase in LPx compared with the AO group (similar values as in OO). The activity of XOD was also increased compared to the AO group, but insignificantly, while it was statistically significantly decreased compared to the OO group. Depending on treatment, bromocriptine caused different activities. The best effects were observed in the AB3 group.

Cahill *et al*. [22] showed that acute alcohol stress causes depletion of the protective effects of antioxidants. Acute alcohol stress inhibits its own metabolism by cytochrome P450 1E2 and, probably, metabolism of some lipophilic xenobiotics (e.g. drugs).

The activity of GSHR is increased only in AB1 group compared to the OO and AO group controls. Other groups do not show a statistically different change in the activity of this enzyme. The activity of Px is increased in all groups compared to the AO controls, in contrast to the OO control group, where it is higher only in the AH and AA group. The increase in CAT activity is statistically significant in all groups in comparison with the OO control and reduced in all groups except AB1 in comparison with the AO control group.

Since CAT activity is highly increased in the AO group compared with the OO group we suggest that an alternative metabolic pathway for the oxidation of ethanol is being turned on that includes the catalytic activity of the enzyme catalase [23]. Reduced CAT activity in groups treated with medications is most probably a consequence of drug metabolism and activity of H_2_O_2,_ in agreement with increased activity of peroxidase in all groups treated with medications. We hypothesize that either the drug itself or its metabolite is being oxidized in the process.

Interestingly the activity of XOD, which is a prooxidative enzyme, is not increased, and in fact is reduced in all groups in comparison with the OO control group. We suggest that acute alcohol stress did not induce this enzyme in time under our experimental conditions.

Inhibition of aldehyde oxidase and xantin oxidase with specific inhibitors lowers the production of alkanes i.e. ethanol induced lipid peroxidation [6,7] in our case; intensity of LPx is reduced in all groups as compared to the OO group and as compared to AO group with the exception of the AA group.

Based on our results we can conclude that the AB1 group shows the greatest protective effect. In this group, the content of GSH is reduced the least as compared to all groups, which corresponds to the activity of the enzymes GSHR and GSHPx. The activity of the prooxidative enzyme XOD and the intensity of lipid peroxidation are reduced as well.

In other groups, it is possible that antioxidative and prooxidative systems displays protective effects and that these medications reduce the effect of alcohol induced stress. Medications used in this study have different pharmacological effects and different structures, but act similarly on the parameters measured in this work and under the conditions used in our experiments.

## Experimental

### General

Investigation was conducted on sexually mature males of laboratory Wistar rats, with average body weight 200-230 grams and age up to 3 months. Rats were bred in the vivarium of the Centre for Biomedical Investigation Galenika a.d. Animals were kept in standard plexiglass cages at constant room temperature 22±1ºC, with circadian rhythm (day/night), and were fed standard laboratory rat feed, produced by the Veterinary Institute in Zemun. The number of rats was 5 per cage. Animals were treated according to the principles of the international declaration Guide for care and Use of Laboratory Animals (NIH publication № 85-23). All animals were exposed to 24-hour fasting prior treatment with alcohol and put in metabolic plexiglass cages with wire floor to prevent coprofagia.

Average single doses of investigated drugs were selected on the basis of human dosage and Clark's formula. Experiments were conducted in the same day interval (8-15 h).

Alcohol stress was induced by intragastric administration of 1 mL of 100% alcohol. Animals were returned to metabolic cages, but no water was given to them. After one hour, animals were sacrificed by ether narcosis. Animals that were not exposed to such stress and were not treated with any drug acted as a control group.

The following drugs were used in the experiments:

Bromocriptine^®^, tablets 2.5 mg, Zdravlje, Leskovac, Serbia. Dissolved in distilled water right before administration.
Haldol^®^decanoate-haloperidol decanoate, 50 mg/mL ampoule, Janssen-Cilag, Division of Johnson & Johnson S.E. d.o.o., Ljubljana, Slovenia.
Hemomycin^®^ - azithromycin, 200 mg/5 mL, Hemofarm, Vrsac, Serbia.

Of the drugs used in this study, we only studied the dose dependency in the case of bromocriptine since this drug has a wide therapeutic window in comparison with the other two.

### Animal treatment

OO group- control – (untreated animals), AO group - alcohol (control without drugs), AB1 group – alcohol + bromocriptine, 24 h n.g. prior to stress 12.5 mg/kg bw. AB2 group – alcohol + bromocriptine n.g. 24 h prior to stress 37.5 mg/kg bw; AB3 group – alcohol + bromocriptine n.g. 3 h prior to stress 25 mg/kg bw; AH group – alcohol + haloperidol i.p. 30 minutes prior to stress 25 mg/kg bw; AA group – alcohol + azithromycin n.g. 250 mg/kg bw during 5 days prior to stress, on the fifth day 2.5 h prior to stress.

### Biochemical assays

Animals were decapitated after treatments and the liver was extracted. The liver was homogenized in a Potter homogenizator with 50 mM TRIS-HCl, 250 mM sucrose in ratio 1:3 at 4^o^C. Obtained homogenates were filtered and the following biochemical parameters were determined:

Extent of lipid peroxidation LPx was determined after Buege and Aust [24], activity of peroxidase (Px) was measured after Simon *et al*. [25], and the effects of catalase (CAT) after Beers and Sizer [26]. Activity of glutathione peroxidase (GSH-Px) was evaluated as described Chin *et al*. [27], xanthine oxidase (XOD) after Bergmayer [28], glutathione reductase (GSHR) after Glatzle and Vuillenmir [29], content of reduced glutathione (GSH) after Kapetanović and Mieyal [30]. Activity of alanine transaminase (ALT) was assayed in the serum using original Bayer Simens bar coded reagents. The content of the total protein was determined after Gornall *et al*. [31].

### Statistical analysis

Results of biochemical analyses are presented as mean value°standards deviation (S.D.). Difference between control and test groups was analyzed by using Student t test (significant difference at p ° 0.05 confidence level). Correlation between the investigated groups was performed using test ONE-WAY ANOVA (one-way variance analysis).
